# Musculoskeletal pain and new-onset poor physical function in elderly survivors of a natural disaster: a longitudinal study after the great East Japan earthquake

**DOI:** 10.1186/s12877-019-1283-z

**Published:** 2019-10-17

**Authors:** Yutaka Yabe, Yoshihiro Hagiwara, Takuya Sekiguchi, Yumi Sugawara, Masahiro Tsuchiya, Nobuyuki Itaya, Shinichirou Yoshida, Yasuhito Sogi, Toshihisa Yano, Takahiro Onoki, Ichiro Tsuji, Eiji Itoi

**Affiliations:** 1 Department of Orthopaedic Surgery, Tohoku University School of Medicine, 2-1 Seiryo-machi, Aoba-ku, Sendai, Miyagi 980-8574 Japan; 2 Division of Epidemiology, Department of Health Informatics and Public Health, Tohoku University Graduate School of Public Health, 2-1 Seiryo-machi, Aoba-ku, Sendai, Miyagi 980-8575 Japan; 3 Department of Nursing, Faculty of Health Science, Tohoku Fukushi University, 1-8-1 Kunimi, Aoba-ku, Sendai, Miyagi 981-8522 Japan

**Keywords:** Great East Japan earthquake, Musculoskeletal pain, Natural disaster, Physically disabled, Survivor

## Abstract

**Background:**

Functional disability is a significant problem after natural disasters. Musculoskeletal pain is reported to increase after disasters, which can cause functional disability among survivors. However, the effects of musculoskeletal pain on functional decline after natural disasters are unclear. The present study aimed to examine the association between musculoskeletal pain and new-onset poor physical function among elderly survivors after the Great East Japan Earthquake.

**Methods:**

A longitudinal study was conducted on survivors aged ≥65 years at three and 4 years after the Great East Japan Earthquake. A total of 747 persons were included in this study. Physical function was assessed using the Kihon Checklist. New-onset poor physical function was defined as low physical function not present at 3 years but present at 4 years after the disaster. Knee, hand or foot, low back, shoulder, and neck pain was assessed using a self-reported questionnaire and was defined as musculoskeletal pain. Musculoskeletal pain at 3 years after the disaster was categorized according to the number of pain regions (0, 1, ≥ 2). Multiple logistic regression analyses were performed to calculate the odds ratio (OR) and 95% confidence interval (95% CI) for new-onset poor physical function due to musculoskeletal pain.

**Results:**

The incidence of new-onset poor physical function was 14.9%. New-onset poor physical function was significantly associated with musculoskeletal pain. Compared with “0” musculoskeletal pain region, the adjusted ORs (95% CI) were 1.39 (0.75–2.58) and 2.69 (1.52–4.77) in “1” and “≥ 2” musculoskeletal pain regions, respectively (*p* for trend = 0.003).

**Conclusions:**

Musculoskeletal pain is associated with new-onset poor physical function among elderly survivors after the Great East Japan Earthquake. Monitoring musculoskeletal pain is important to prevent physical function decline after natural disasters.

## Background

Functional disability is a significant problem after natural disasters, especially among elderly survivors [1]. The Great East Japan Earthquake (GEJE) and tsunami destroyed the northeast coastal region of Japan in 2011. Due to this serious disaster, approximately 18,500 people died or went missing and 400,000 buildings were destroyed [2]. Reconstruction after disasters of this magnitude takes a significant amount of time; 73,000 survivors continue to take refuge from their hometowns and 13,000 people have lived in prefabricated temporary houses for at least 7 years after the GEJE [3]. Tomata et al., in a longitudinal study after the GEJE, reported that the incidence of functional disability was higher in the disaster areas than in non-disaster areas [4]. Preventing functional decline is an important issue after natural disasters and should be considered even during the disaster recovery period. The environmental habitat changed and many evacuees lost connections with their local communities after the GEJE [1, 5]. Because older survivors are more vulnerable to these changes, they have had fewer opportunities to go out into the community. Many have become homebound [6, 7], which is generally considered a predictor of functional decline [8]. Furthermore, some authors have reported other factors related to functional disability after the GEJE. Survivors living in temporary housing have limited physical activity, which can lead to functional decline [7]. The rate of psychological distress was reported to increase after the GEJE [9], which was associated with functional disability in older survivors [10]. Higher rates of musculoskeletal pain after the GEJE have also been reported [11–13], which can cause functional disability in survivors. However, the effect of musculoskeletal pain on functional decline after natural disasters has not yet been reported.

The present study aimed to examine the association between musculoskeletal pain and new-onset poor physical function among elderly survivors in the recovery period after the GEJE.

## Methods

### Participants

**Fig. 1:**
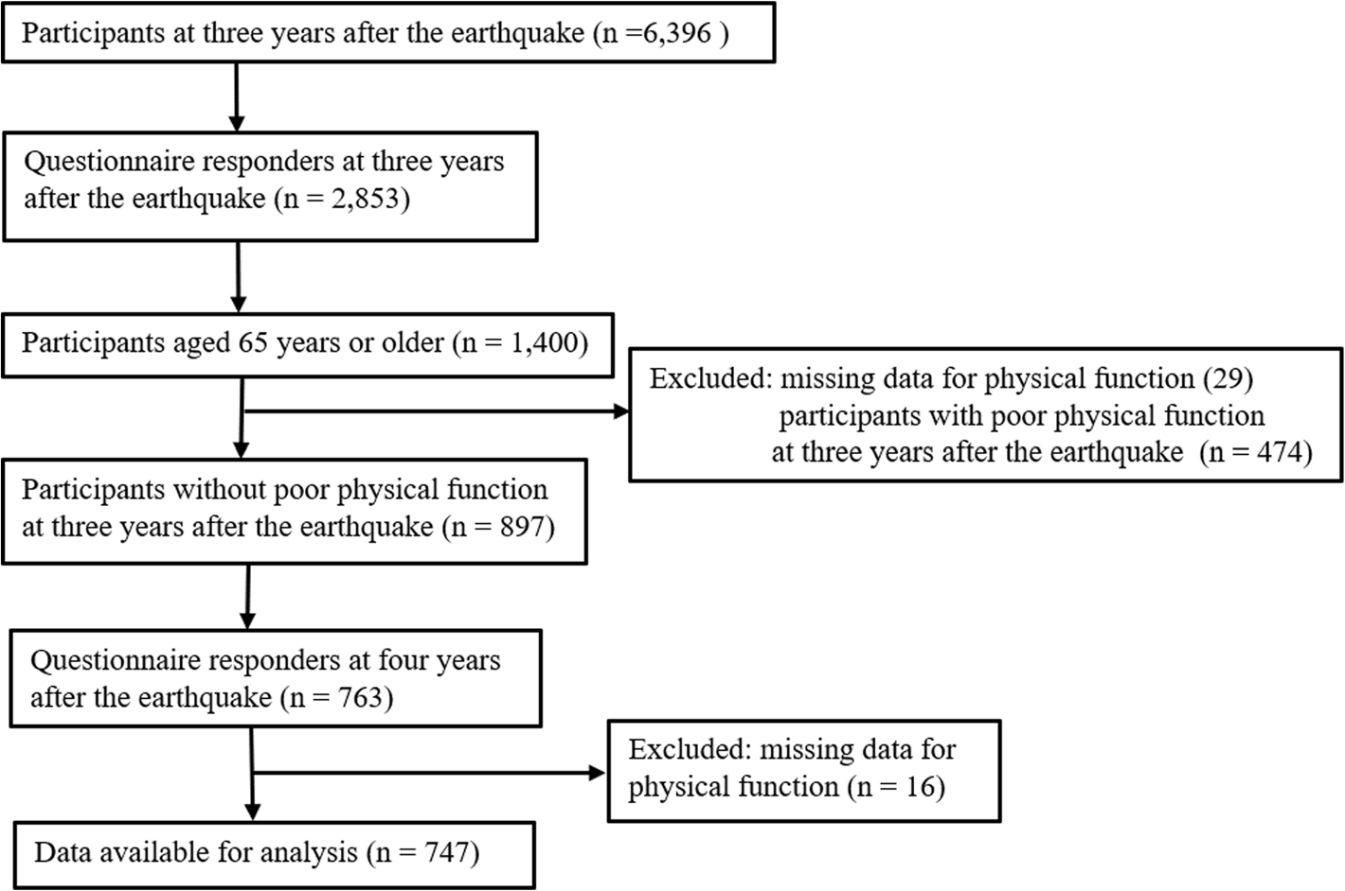
Flowchart of the present analysis

A longitudinal study was conducted on GEJE survivors living in seriously damaged disaster areas, including Ogatsu and Oshika districts in Ishinomaki City and Wakabayashi Ward in Sendai City, Miyagi Prefecture. We analyzed the data of surveys conducted at three and 4 years after the disaster. The surveys were examined every 6 months starting at 3 months after the GEJE using a self-reported questionnaire. The first study was conducted with residents registered in the Residential Registry of Ogatsu and Oshika districts and residents living in prefabricated houses in Wakabayashi Ward. For 3 years after the disaster, survivors (aged 18 years or over) who were registered in the Residential Registry of Ogatsu and Oshika districts and who participated in the previous survey in the Wakabayashi Ward were recruited (*n* = 6396). Altogether, there were 2853 (44.6%) participants. Among those, 1400 were aged 65 years and over. We excluded participants who already had poor physical function (*n* = 474) or for whom data on physical function were missing (*n* = 29). Study participants were followed up at 4 years after the disaster. The follow-up rate in this period was 85.1% (763/897); 16 persons were excluded due to missing data on physical function. Finally, 747 persons were analyzed in this study (Fig. 1).

### Outcome variables

Physical function was assessed using the physical function score from the Kihon Checklist (KCL), which is a self-reported questionnaire consisting of five yes/no questions. New-onset poor physical function was defined as poor physical function that was absent at 3 years but present at 4 years after the disaster. The questions were as follows: “Can you climb stairs without holding onto a handrail or wall?,” “Can you get up from a chair without grabbing something?,” “Are you able to walk for about 15 minutes?,” “Have you fallen in the past year?,” and “Are you very worried about falling?” [14]. Each negative response received one point, and poor physical function was defined by a score of ≥3/5 in the physical function category on the KCL [14, 15].

### Main predictor

Musculoskeletal pain at 4 years after the disaster was evaluated using self-reported questionnaires based on the Comprehensive Survey of Living Conditions [3]. The questions included: “Have you had symptoms in the last few days? If yes, please place a check mark next to all your symptoms.” The examples of choices were palpitations, dizziness, diarrhea, gastric pain, and musculoskeletal symptoms such as knee, hand or foot, low back, shoulder, and neck pain [16]. Among those, knee, hand or foot, low back, shoulder, and neck pain was defined as musculoskeletal pain [17]. Furthermore, musculoskeletal pain was categorized according to the number of pain regions (0, 1, and ≥ 2) [12, 18].

### Covariates

The following variables were considered as covariates according to a previous report [13]: sex, age, body mass index, living areas, smoking habits, drinking habits, chronic conditions (hypertension, diabetes mellitus, myocardial infarction, and cerebral stroke), working status, walking time per day, living environment, subjective economic hardship, psychological distress, sleep disturbance, and social isolation at 3 years after the disaster. Psychological distress, sleep disturbance, and social isolation were assessed and defined as a score ≥ 10/24 on the Kessler Psychological Distress Scale [19], ≥ 6/24 on the Athens Insomnia Scale [20], and < 12/30 on the Lubben Social Network Scale [21], respectively.

### Statistical analysis

Variables are presented as frequencies and percentages (%). Crude and multiple logistic regression analyses were performed to calculate odds ratios (ORs) and 95% confidence intervals (95% CIs) for new-onset poor physical function according to musculoskeletal pain at 3 years after the disaster. Variables used in the analysis were sex, age (< 75 or ≥ 75 years), body mass index (< 18.5, 18.5–24.9, ≥ 25, or unknown), living areas (Ogatsu, Oshika, or Wakabayashi), smoking habits (non-smoking, smoking, or unknown), drinking habits (non-drinking, < 45.6 g of alcohol/day, ≥ 45.6 g of alcohol/day, or unknown), chronic conditions (absence or presence of hypertension, diabetes mellitus, myocardial infarction, and cerebral stroke), working status (unemployed, employed, or unknown), and walking time per day (< 0.5 h, 0.5 to < 1 h, ≥ 1 h, or unknown) (Model 1). Furthermore, variables related to disasters, such as living environment (same house as before the GEJE, prefabricated house, new house, others, or unknown), subjective economic hardship (normal, a little bit hard, hard, very hard, or unknown), psychological distress (absence, presence, or unknown), sleep disturbance (absence, presence, or unknown), and social isolation (absence, presence, or unknown), were added (Model 2). The ORs and 95% CIs for new-onset poor physical function according to each musculoskeletal pain region were also evaluated. Moreover, we divided the participants into two subgroups according to age (< 75 [*n* = 456], or ≥ 75 [*n* = 291] years), and ORs and 95% CIs for new-onset poor physical function were assessed in the same manner. SPSS version 24.0 (SPSS Japan Inc., Tokyo, Japan) was used for all analyses and differences at *p* < 0.05 were considered statistically significant.

## Results

The participants’ baseline characteristics are presented in Table 1. Of the 747 participants analyzed, 489 (65.5%), 126 (16.9%), and 132 (17.7%) had “0,” “1,” and “≥ 2″ musculoskeletal pain regions, respectively (Table 1). New-onset poor physical function had an incidence rate of 14.9% (111/747) and was significantly associated with musculoskeletal pain. Compared with “0″ musculoskeletal pain region, the adjusted ORs (95% CI) were 1.49 (0.83–2.65) and 2.62 (1.55–4.42) in “1″ and “≥2″ musculoskeletal pain regions, respectively, in Model 1 (p for trend = 0.001) and were 1.39 (0.75–2.58) and 2.69 (1.52–4.77) in “1″ and “≥2″ musculoskeletal pain regions, respectively, in Model 2 (p for trend = 0.003) (Table 2). For each musculoskeletal pain region, new-onset poor physical function was associated with knee and hand or foot pain, but not with low back, shoulder, and neck pain. The adjusted ORs (95% CI) for new-onset poor physical function were 2.51 (1.43–4.40) for knee pain, 2.60 (1.44–4.71) for hand or foot pain, 1.61 (0.94–2.78) for low back pain, 1.77 (0.78–4.04) for shoulder pain, and 1.50 (0.81–2.78) for neck pain (Table 3).

**Table 1:** Baseline characteristics of the participants according to the number of musculoskeletal pain sites

		Number of musculoskeletal pain sites, *n* (%)			
		Total	0	1	≥ 2
		747	489	126	132
Sex	Male	382 (51.1)	266 (54.4)	54 (42.9)	62 (47.0)
	Female	365 (48.9)	223 (45.6)	72 (57.1)	70 (53.0)
Age	< 75	456 (61.0)	305 (62.4)	68 (54.0)	83 (62.9)
	≥ 75	291 (39.0)	184 (37.6)	58 (46.0)	49 (37.1)
BMI^a^	< 18.5	18 (2.4)	13 (2.7)	3 (2.4)	2 (1.5)
	18.5–24.9	426 (57.0)	290 (59.3)	71 (56.3)	65 (49.2)
	≥ 25	279 (37.3)	174 (35.6)	44 (34.9)	61 (46.2)
Living area	Ogatsu	349 (46.7)	236 (48.3)	53 (42.1)	60 (45.5)
	Oshika	274 (36.7)	180 (36.8)	50 (39.7)	44 (33.3)
	Wakabayashi	124 (16.6)	73 (14.9)	23 (18.3)	28 (21.2)
Smoking habits^a^	Non-smoking	602 (80.6)	394 (80.6)	103 (81.7)	105 (79.5)
	Smoking	80 (10.7)	54 (11.0)	10 (7.9)	16 (12.1)
Drinking habits^a^	Non-drinking	430 (57.6)	278 (56.9)	79 (62.7)	73 (55.3)
	< 45.6 g of alcohol/day^b^	161 (21.6)	101 (20.7)	25 (19.8)	35 (26.5)
	≥ 45.6 g of alcohol/day^b^	52 (7.0)	41 (8.4)	4 (3.2)	7 (5.3)
Chronic conditions	Hypertension	423 (56.6)	260 (53.2)	71 (56.3)	92 (69.7)
	Diabetes mellitus	97 (13.0)	58 (11.9)	16 (12.7)	23 (17.4)
	Myocardial infarction	71 (9.5)	38 (7.8)	12 (9.5)	21 (15.9)
	Cerebral stroke	8 (1.1)	3 (0.6)	2 (1.6)	3 (2.3)
Working status^a^	Unemployed	522 (69.9)	341 (69.7)	91 (72.2)	90 (68.2)
	Employed	202 (27.0)	133 (27.2)	31 (24.6)	38 (28.8)
Walking time/day^a^	≥ 1 h	205 (27.4)	153 (31.3)	29 (23.0)	23 (17.4)
	0.5 to < 1 h	30 (44.2)	218 (44.6)	56 (44.4)	56 (42.4)
	< 0.5 h	198 (26.5)	108 (22.1)	40 (31.7)	50 (37.9)
Living environment^a^	Same house as before the GEJE	219 (29.3)	145 (29.7)	43 (34.1)	31 (23.5)
	Prefabricated house	295 (39.5)	190 (38.9)	52 (41.3)	53 (40.2)
	New house	109 (14.6)	70 (14.3)	12 (9.5)	27 (20.5)
	Others	123 (16.5)	83 (17.0)	19 (15.1)	21 (15.9)
Subjective economic hardship^a^	Normal	372 (49.8)	262 (53.6)	46 (36.5)	64 (48.5)
	A little bit hard	206 (27.6)	132 (27.0)	40 (31.7)	34 (25.8)
	Hard	115 (15.4)	60 (12.3)	35 (27.8)	20 (15.2)
	Very hard	34 (4.6)	16 (3.3)	4 (3.2)	14 (10.6)
Psychological distress^a^	Absence	651 (87.1)	442 (90.4)	99 (78.6)	110 (83.3)
	Presence	61 (8.2)	25 (5.1)	17 (13.5)	19 (14.4)
Sleep disturbance^a^	Absence	552 (73.9)	401 (82.0)	82 (65.1)	69 (52.3)
	Presence	185 (24.8)	80 (16.4)	43 (34.1)	62 (47.0)
Social isolation^a^	Absence	579 (77.5)	383 (78.3)	95 (75.4)	101 (76.5)
	Presence	164 (22.0)	102 (20.9)	31 (24.6)	31 (23.5)

^a^Because each item has a limited number of respondents, the actual number is not necessarily in accordance with the total.

^b^22.8 g of alcohol amount to 1 go or traditional unit of sake (180 ml), which also approximates to two glasses of wine (200 ml), or beer (500 ml) in terms of alcohol content.

categorical variables are presented as numbers and percentage (%).

**Table 2:** Influence of musculoskeletal pain on new-onset poor physical function

	Number of musculoskeletal pain sites				
	total	0	1	≥ 2	P for trend
Participants	747	489	126	132	
New-onset poor physical function, n (%)	111 (14.9)	54 (11.0)	22 (17.5)	35 (26.5)	
Crude OR (95% CI)		1	1.70 (0.99–2.92)	2.91 (1.80–4.69)	< 0.001
Model 1 OR (95% CI)		1	1.49 (0.83–2.65)	2.62 (1.55–4.42)	0.001
Model 2 OR (95% CI)		1	1.39 (0.75–2.58)	2.69 (1.52–4.77)	0.003

**Table 3:** Influence of each musculoskeletal pain on new-onset poor physical function

		absence	presence	*P* value
Knee pain	Participants	641	106	
	Crude OR (95% CI)	1	2.73 (1.68–4.42)	< 0.001
	Adjusted OR (95% CI)	1	2.51 (1.43–4.40)	0.001
Hand or foot pain	Participants	653	94	
	Crude OR (95% CI)	1	2.73 (1.65–4.51)	< 0.001
	Adjusted OR (95% CI)	1	2.60 (1.44–4.71)	0.002
Low back pain	Participants	617	130	
	Crude OR (95% CI)	1	1.99 (1.24–3.18)	0.004
	Adjusted OR (95% CI)	1	1.61 (0.94–2.78)	0.085
Shoulder pain	Participants	704	43	
	Crude OR (95% CI)	1	2.08 (1.01–4.25)	0.046
	Adjusted OR (95% CI)	1	1.77 (0.78–4.04)	0.175
Neck pain	Participants	642	105	
	Crude OR (95% CI)	1	1.77 (1.06–2.95)	0.03
	Adjusted OR (95% CI)	1	1.50 (0.81–2.78)	0.20

In the stratified analyses, new-onset poor physical function was associated with the presence of musculoskeletal pain in both age groups. Compared with “0” pain region, the adjusted ORs (95% CI) for new-onset poor physical function were 2.63 (1.04–6.63) for “1” pain region and 2.74 (1.16–6.48) for “≥ 2” pain regions in the age < 75 group (p for trend = 0.031) and were 1.06 (0.40–2.81) for “1” pain region and 2.99 (1.28–6.96) for “≥ 2” pain regions in the age ≥ 75 group (p for trend = 0.029) (Table 4).

**Table 4:** Stratified analysis for each age group

	Number of musculoskeletal pain sites				
	total	0	1	≥ 2	P for trend
Age < 75					
Participants	456	305	68	83	
New-onset poor physical function, n (%)	48 (10.5)	21 (6.9)	11 (16.2)	16 (19.3)	
Crude OR (95% CI)		1	2.61 (1.19–5.71)	3.23 (1.60–6.52)	0.002
Adjusted OR (95% CI)		1	2.63 (1.04–6.63)	2.74 (1.16–6.48)	0.031
Age ≥ 75					
Participants	291	184	58	49	
New-onset poor physical function, n (%)	63 (21.6)	33 (17.9)	11 (19.0)	19 (38.8)	
Crude OR (95% CI)		1	1.07 (0.50–2.28)	2.90 (1.46–5.76)	0.008
Adjusted OR (95% CI)		1	1.06 (0.40–2.81)	2.99 (1.28–6.96)	0.029

Adjusted for sex, age, body mass index, living area, smoking habits, drinking habits, complications, working status, and walking time (Model1). Additionally, adjusted for living status, subjective economic hardship, psychological distress, sleep disturbance, and social isolation (Model 2).

*OR* Odds Ratio, *CI* Confidence Interval.

Adjusted for sex, age, body mass index, living area, smoking habits, drinking habits, chronic conditions, working status, walking time, living status, subjective economic hardship, psychological distress, sleep disturbance, and social isolation.

*OR* odds ratio, *CI* confidence interval.

Adjusted for sex, body mass index, living area, smoking habits, drinking habits, chronic conditions, working status, walking time, living status, subjective economic hardship, psychological distress, sleep disturbance, and social isolation.

*OR* odds ratio, *CI* confidence interval.

## Discussion

This study showed that musculoskeletal pain is associated with new-onset poor physical function among elderly survivors in the recovery period of the GEJE. There have been some reports of functional disabilities after natural disasters [1, 4, 6, 10, 14]. Some factors related to disasters, such as living in temporary housing [14] and psychological distress [10], are reported to be associated with functional decline; nonetheless, to our knowledge, this is the first study to show that musculoskeletal pain led to poor physical function after a natural disaster. Some authors have reported an association between musculoskeletal pain and physical function in community-dwelling elderly [18, 22, 23]. Pain induced by musculoskeletal disorders, such as osteoarthritis and low back pain, limits physical function [23]. Musculoskeletal pain also reduces the ability to perform physical activities and causes dis-use that can result in muscle weakness, reduced joint range of motion, and reflex inhibition of skeletal muscles that can lead to gait instability or falls [18, 22]. Some authors have shown that multi-site pain is associated with functional disability among the elderly [22, 24]. Furthermore, Eggermont et al. reported that the association between musculoskeletal pain and functional disability became stronger as the number of pain sites increased [25]. This is similar to our results. Natural disasters deprive numerous lives and properties from survivors, changing their living status and economic condition [5, 13] and resulting in psychological distress [17], sleep disturbance [12], and social isolation [21]. These conditions can have negative effects on the functional condition of survivors. Nevertheless, the association between musculoskeletal pain and new-onset physical function was similar after adjustment for these factors. Preceding musculoskeletal pain is associated with functional decline, even in special circumstances such as natural disasters. After the GEJE, psychological distress, sleep disturbance, and economic hardship increased among survivors [9, 26]. These factors are thought to lead to increased musculoskeletal pain [13, 27–30]. Approximately 34.5% of survivors had musculoskeletal pain and more than half of them had multi-site pain in this study. Functional disabilities increased after the GEJE [4] and increased musculoskeletal pain could be one of the main reasons for it. Furthermore, the association between musculoskeletal pain and new-onset poor physical function was different according to each pain region; in particular, it was significant for knee and hand or foot pain, but not for low back, shoulder, and neck pain. It is possible that this result depended on the specific questionnaire used in assessing physical function in this study. Physical function scores on the KCL focused on the importance of walking ability and falls [14], which can be easily affected by lower extremity pain. Leveille et al. reported that only foot pain was related to an increased risk of falls in site-specific pain [31]. Yiengprugsawan et al. showed that the impact of site-specific pain on activities of daily living related to mobility was stronger with knee pain than with low back pain [24]. Lower extremity pain by itself was considered to affect the onset of poor physical function among survivors by lowering walking ability or increasing the risk of falls.

The prevalence of new-onset poor physical function among survivors aged ≥75 years was approximately twice the rate of that among survivors aged < 75 years. Functional decline was more common in older survivors, which is in accordance with the former report after the GEJE [1]. In the age < 75 group, both single-site and multi-site musculoskeletal pains were associated with new-onset poor physical function. On the other hand, the association was significant when the number of pain sites was ≥2 in the age ≥ 75 group. Because aging is associated with structural and functional changes [32], various factors affect functional decline in the elderly, which may minimize the effect of single-site musculoskeletal pain on physical function among survivors aged ≥75 years. In both groups, musculoskeletal pain was associated with new-onset poor physical function. It is important to take note of musculoskeletal pain, particularly multi-site pain, to prevent physical function decline among survivors of natural disasters. Hasegawa et al. reported that a community-based exercise class was effective for reducing musculoskeletal pain [33]. It is important for elderly survivors to maintain physical activity levels to reduce musculoskeletal pain, which can prevent physical function decline.

## Limitations

This study has several limitations. First, the questionnaires and informed consent forms were mailed and the response rate was not high. The people who responded might have been in better health than those who did not respond, which could affect the results. Second, musculoskeletal pain and physical function were assessed in only two periods and changes in other periods were unknown. Third, musculoskeletal pain was assessed using self-reported questionnaires based on the Comprehensive Survey of Living Conditions. Although this survey is widely accepted in Japan, the reliability and validity of this method were not evaluated in this study. Furthermore, the questionnaires included five pain sites but did not include other musculoskeletal pains such as hip or elbow pain. Because these pains could have also led to physical function decline, this might have affected the results. In addition, the degree of pain was not assessed. Finally, this study did not include a control group, because the disaster struck a vast area of Japan.

## Conclusion

Musculoskeletal pain is associated with new-onset poor physical function among elderly survivors in the recovery period after the GEJE. Monitoring musculoskeletal pain is important to prevent physical function decline after natural disasters.

## Data Availability

All relevant data are contained in this article.

## Abbreviations

95% CI: 95% confidence interval
GEJE: Great East Japan Earthquake
KCL: Kihon Checklist
OR: Odds ratio
